# Constructing a framework for risk analyses of climate change effects on the water budget of differently sloped vineyards with a numeric simulation using the Monte Carlo method coupled to a water balance model

**DOI:** 10.3389/fpls.2014.00645

**Published:** 2014-12-10

**Authors:** Marco Hofmann, Robert Lux, Hans R. Schultz

**Affiliations:** Institut für Allgemeinen und ökologischen Weinbau, Hochschule Geisenheim UniversityGeisenheim, Germany

**Keywords:** climate change, grapevine, model, radiation interception, sap flow, soil water budget, steep slope, vine transpiration

## Abstract

Grapes for wine production are a highly climate sensitive crop and vineyard water budget is a decisive factor in quality formation. In order to conduct risk assessments for climate change effects in viticulture models are needed which can be applied to complete growing regions. We first modified an existing simplified geometric vineyard model of radiation interception and resulting water use to incorporate numerical Monte Carlo simulations and the physical aspects of radiation interactions between canopy and vineyard slope and azimuth. We then used four regional climate models to assess for possible effects on the water budget of selected vineyard sites up 2100. The model was developed to describe the partitioning of short-wave radiation between grapevine canopy and soil surface, respectively, green cover, necessary to calculate vineyard evapotranspiration. Soil water storage was allocated to two sub reservoirs. The model was adopted for steep slope vineyards based on coordinate transformation and validated against measurements of grapevine sap flow and soil water content determined down to 1.6 m depth at three different sites over 2 years. The results showed good agreement of modeled and observed soil water dynamics of vineyards with large variations in site specific soil water holding capacity (SWC) and viticultural management. Simulated sap flow was in overall good agreement with measured sap flow but site-specific responses of sap flow to potential evapotranspiration were observed. The analyses of climate change impacts on vineyard water budget demonstrated the importance of site-specific assessment due to natural variations in SWC. The improved model was capable of describing seasonal and site-specific dynamics in soil water content and could be used in an amended version to estimate changes in the water budget of entire grape growing areas due to evolving climatic changes.

## Introduction

Grapevines are cultivated on 6 out of 7 continents, between latitudes 4° and 51° in the Northern Hemisphere (NH) and between 6° and 45° in the Southern Hemisphere (SH) across a large diversity of climates (Tonietto and Carbonneau, 2004). Accordingly, the range and magnitude of environmental factors and the principal environmental constraints differ considerably from region to region. Wine grapes are traditionally grown in geographical regions where the growing season (April–October for the NH) mean temperature is within the range of 12–22°C (Jones, 2006). Warming during the growing season has been observed in all studied wine regions over the past 50–60 years (i.e., Schultz, 2000; Jones et al., 2005a; Webb et al., 2007, 2011; Santos et al., 2012). Observed and predicted changes in temperature have a pronounced effect on the geographical distribution of where grapevines can be grown (Kenny and Harrison, 1992; Jones et al., 2005b; Schultz and Jones, 2010; Santos et al., 2012). Observed advancement in phenological events and specifically maturity have recently also been correlated to a continuous reduction in soil water content as a co-factor to temperature (Webb et al., 2012). Within the existing production areas, water shortage is probably the most dominant environmental constraint (Williams and Matthews, 1990) and even in moderate temperate climates, grapevines often face some degree of drought stress during the growing season (Morlat et al., 1990; van Leeuwen and Seguin, 1994; Gaudillère et al., 2002; Gruber and Schultz, 2005).

Recent projections for the major world grape growing areas using various model approaches driven by 17 global climate models (GCMs) projected substantial reductions in suitable area for Viticulture largely due to changes in water availability related to shifts in precipitation rate and/or distribution, increases in evaporative demand and in many cases reduced access to water for irrigation (Hannah et al., 2013). Most European grape growing areas are non-irrigated and there is a rising concern if this is sustainable in the future. Additionally, many of the most valuable areas in terms of quality and reputation are located on steep slopes which may exacerbate the impact of climate change due to a reduced potential for adaptation (high labor costs, technical challenges, access to water a.s.o). Southern Germany has many examples for these landscapes since wine-growing regions are mainly located in river valleys where Viticulture has been practized on steep slopes for hundreds sometimes several thousand years (Weeber, 1993). Mean annual precipitation (530–750 mm) is generally low in these regions and soil water holding capacity (SWC) is very heterogeneous, with the percentage of vineyards with low SWC being relatively high (example Rheingau region; SWC < 125 mm for nearly 50% of steep slope areas, Löhnertz et al., 2004). Therefore, risk assessment of possible consequences of climate change on soil and plant water budget needs to be on a finer scale and requires a functional plant or vineyard model, respectively, which can be scaled up from vineyard plots to entire regions.

There are several approaches which have been taken previously to model the water budget of vineyards and the use of crop coefficients is the most widely spread (Allen et al., 1998). However, grapevine canopies represent a large array of possible structures (shape, leaf/fruit/stem distribution, density) imbedded in an equally large spectrum of possible vineyard geometries (distances between and within rows) which in conjunction with a variety of management practices and soil properties affect vineyard transpiration and render the use of standard crop coefficients (*K*_*c*_) difficult (Williams and Ayars, 2005; Fandiño et al., 2012). These difficulties are also reflected in other approaches based on the Shuttleworth and Wallace (1985) model which could separate between vine transpiration and soil evaporation, by applying individual evapotranspiration controlling resistances to plants or the soil and combining one dimensional models of crop transpiration and soil evaporation. This model proved to be very sensitive to the parameterization of the leaf area index (LAI, used to model net energy separation) and canopy resistance, and might be combined with a more detailed model to separate net radiation on plants or soil in order to apply the model to complete growing regions (Ortega-Farias et al., 2007, 2010; Poblete-Echeverría and Ortega-Farias, 2009).

There has been substantial progress in the description of grape canopy structure and its effect on light interception using two-dimensional modeling (Schultz, 1995) and later three-dimensional digitizing technology (Mabrouk et al., 1997; Sinoquet et al., 1998; Louarn et al., 2007) which consequently lead to the development of complex three-dimensional models of plant architecture on an organ scale (Louarn et al., 2008a; López-Lozano et al., 2009; Iandolino et al., 2013). Beside of many applications of functional-structural models of this detail in assessing plant architecture effects on radiative transfer and whole plant gas exchange (Louarn et al., 2008b; López-Lozano et al., 2011; Prieto et al., 2012) they remain difficult to parameterize and have not yet been scaled up to asses for vineyard water use. Lebon et al. (2003) have used a somewhat intermediate approach between simplistic and highly complex to describe the light interception inside of a vineyard in order to separate the evapotranspiration fluxes of grapevines or bare soil and validated the model for different vineyard sites. Celette et al. (2010) extended the model to account for changes in water use by the presence of cover crops. In principle, the model goes back to a geometrical vineyard model of radiation interception and distribution proposed by Riou et al. (1989) with the basic assumption that these are the key drivers of transpiration and evaporation. The model was then extended to include soil water reservoirs (Riou et al., 1994), to account for the feedback of water stress on transpiration (Lebon et al., 2003), to simulate meaningful physiological plant parameters describing the level of water deficit such as predawn water potential (Schultz and Lebon, 2005) and to characterize the radiative balance within important parts of a vineyard canopy such as the fruiting zone (Pieri, 2010a,b). However, the model has never been used to describe radiation interception, and consequently, the water budget in sloped vineyards, where slope and azimuth in conjunction with the degree of latitude have substantial impact on the received solar radiation (Geiger, 1980) and their partitioning on vines or soil, nor has it been coupled to regionalized climate models in order to project changes in vineyard water balance possibly brought about by climate change under these situations.

We therefore had several key objectives:

1. to improve the canopy-structure module;
2. to adapt the model environment so that different degrees of slope and azimuth can be accounted for;
3. to validate the model on different sites against sap flow and soil moisture data and;
4. to use the model in conjunction with several regionalized climate models to project changes in soil and plant water budget for different vineyard sites for the period of the current century.

## Materials and methods

### Vineyard site description

**Figure 1:**
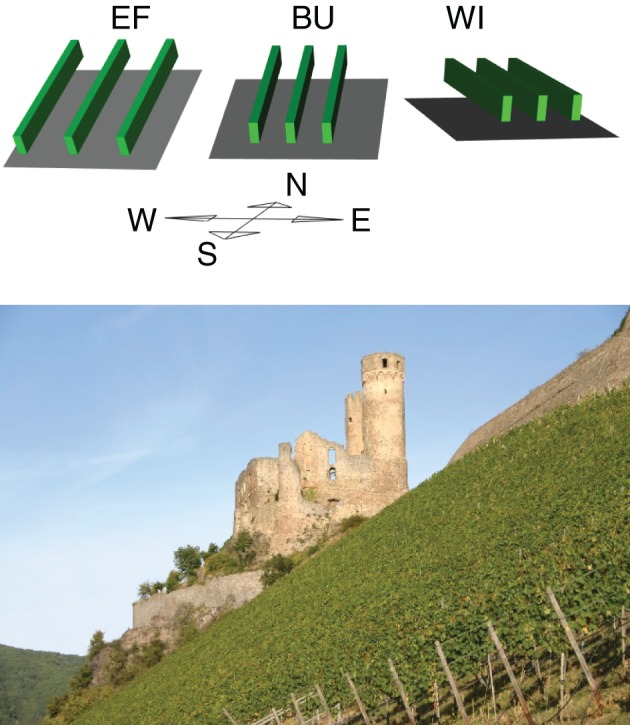
**Upper part:** Graphical outline of three experimental vineyards true to scale in row distance, canopy height and width, slope and aspect ratios. Green cuboids illustrate grapevine rows, soil is gray. **Bottom part**: The experimental vineyard EF with the castle ruin Ehrenfels in the background.

Three commercial vineyards located near Rüdesheim (49°58′N, 7°55′E) with different soil water holding capacities, management practices, canopy geometries and differences in the degree of slope and azimuth were chosen as validation sites for the model (Figure 1). The plots were named Ehrenfels (EF), Burgweg (BU), and Wilgert (WI), planted with *Vitis Vinifera* cv. “Riesling” and trained to a cane or spur pruned VSP Trellis system. The geometry of the canopy (Table 1) was conserved after bloom (mid-June) by hedging two or three times during the summer.

**Table 1:** **Main characteristics of the three commercial vineyards used in the study (azimuth angles east of south are negative and west of south positive)**.

**Site**	**Planting density (vines/ha)**	**Total transpirable soil water (mm), max. depth 1.60 m**	**Canopy height (m)**	**Canopy width (m)**	**Row distance (m)**	**Porosity (min.)**	**Slope/azimuth**	**Fraction of soil covered by vegetation**
EF	4400	85	1.00	0.40	2.50	0.40	35°/8°	0.84
BU	6875	115	1.10	0.40	1.60	0.25	27°/4°	0.40
WI	6875	160	1.35	0.40	1.60	0.25	15°/−21°	0.75

BU and WI were planted in 1983 and grafted onto the rootstock 5C and EF was planted in 1996 and grafted onto Börner. Vineyards EF and BU were on steep slopes (Table 1) with shallow stony soils (<1.5 m depth), poor in loess-loam on largely carbonate-free bedrock (class I, Löhnertz et al., 2004), whereas the soil of WI was medium deep (>1.5 m) with a high proportion of loam and hence a higher water holding capacity than EF and BU (class II, Löhnertz et al., 2004).

All soils were partly covered by a natural population of cover crops and weeds (mainly grasses) whereby the surface area fractions occupied by these plants (*f*_*cc*_) differed between sites (Table 1). A strip of approximately 0.4 m width beneath the vines was kept bare in all plots through the use of herbicides. In EF and WI the soil of each row was covered by cover crops whereas in BU alternating rows were kept free of vegetation by frequent tillage. Inter-row vegetation was kept short through frequent mowing in all vineyards. These types of soil management are representative for many German steep slope wine regions.

### Radiation partitioning model

The original model of Lebon et al. (2003) calculated the amount of radiation absorbed by the vineyard and partitioned this to soil and canopy. The geometry of the canopy was described by the distance between the rows, *D*, and the width, *L*, and the height, *H*, of the grapevine foliage (see Table 2 for a list of symbols). Height and width of the canopy composed a cuboid, whose third edge length corresponded to the length of the grapevine row and was considered infinite. Further input variables were the perpendicular porosity of the vertical foliage walls, the soil surface and leaf albedos, the incoming direct and diffuse solar radiation and the direction of the direct solar radiation. The horizontal faces bordering the top and the bottom of the foliage were considered opaque.

**Table 2:** **List of symbols and abbreviations used**.

*a_dif,v_*	Intercepted fraction of diffuse solar radiation by the vines
*a_v_, a_s_*	Intercepted fraction of direct solar radiation by the vines or the soil, respectively
*E*	Evaporation (lm^−2^day^−1^)
*E_s_*	Evaporation of the vineyard (lm^−2^day^−1^)
*E*_0_	Potential soil evaporation (lm^−2^day^−1^)
*ET_a_*	Actual evapotranspiration of the vineyard (lm^−2^day^−1^)
*ET_a,cc_*	Evapotranspiration of the cover crops (lm^−2^day^−1^)
*ET*_0_	Potential evapotranspiration (horizontal equivalent) (lm^−2^day^−1^)
*ET*_0*s*_	Potential evapotranspiration of the soil surface (lm^−2^day^−1^)
*FTSW*	Fraction of transpirable soil water
*FTSWcc*	Fraction of transpirable soil water accessible by cover crops
*f_cc_*	Surface area fraction covered by cover crops or weeds (constant parameter depending on management practices)
*f_g_*	Ground cover coefficient
*f_R,v_ f_R,s_*	Relative fractions of absorbed radiation by grapevines or soil
*D*	Distance between vine rows (m)
*H*	Height of the grapevine foliage (without stem height) (m)
*I*	Radiant flux density (Wm^−2^)
*k_c,v_*	Grapevine transpiration coefficient
*K_e_*	Soil evaporation coefficient
*K_r_*	Soil evaporation reduction coefficient
*k_s_*	Water stress coefficient
*k_s,cc_*	Cover crop water coefficient
*k_s,v,cc_*	Water extraction coefficient (considers the water extraction of grapevines from the cover crops reservoir)
*L*	Width of the grapevine foliage (m)
LAI	Leaf area index
*L_e_*	Radiance (Wm^−2^sr^−1^)
*N*	Number of emitted or absorbed photons in a numerical Monte Carlo simulation
*p*	Porosity of the foliage (probability for no interception of a photon)
*P*	Precipitation (lm^−2^)
*p_FTSW_*	Threshold value for *FTSW*
*R*_0_	Extraterrestrial radiation (Wm^−2^)
*R_dif_*	Diffuse solar radiation (Wm^−2^)
*R_dir_*	Direct solar radiation (Wm^−2^)
*R_dif,v_*	Diffuse solar radiation absorbed by the grapevine canopy (Wm^−2^)
*R_glob_*	Global solar radiation (Wm^−2^)
*R_s_*	Radiation absorbed by the soil (Wm^−2^)
*R_v_*	Radiation absorbed by the grapevines (Wm^−2^)
*R_vy_*	Radiation absorbed by the vineyard (Wm^−2^)
*REW*	Readily evaporable water (lm^−2^)
*S*	Height of the foliage above ground (stem height)
*SWC*	Soil water holding capacity (lm^−2^ and rooting depth)
*T*_0,v_	Potential grapevine transpiration (lm^−2^day^−1^)
*T_a,v_*	Actual grapevine transpiration (lm^−2^day^−1^)
*TEW*	Total evaporable water (lm^−2^)
*TSW*	Transpirable soil water (lm^−2^)
*TSW_cc_*	Transpirable soil water (accessible by cover crops) (lm^−2^)
*TSW_r_*	Transpirable water of the remaining (non-cover crop) reservoir (lm^−2^)
*TTSW*	Total transpirable soil water (lm^−2^)
*TTSW_cc_*	Total transpirable soil water (accessible by cover crops) (lm^−2^)
*TTSW_r_*	Total transpirable soil water of the remaining (non-cover crop) reservoir (lm^−2^)
*VPD*	Vapor pressure deficit
α_*v*_, α_*s*_	Absorptance of the grapevine foliage or the soil (for single photons)
β	Slope angle of the vineyard
γ	Vineyard azimuth angle (the aspect of the vineyard)
γ_*s*_	Solar azimuth angle
γ_*v*_	Vineyard solar azimuth angle
θ	Angle of incidence (angle between direct radiation beam and the normal to the surface of the vineyard)
θ_*z*_	Zenith angle of the sun
ρ_*s*_, ρ_*l*_	Shortwave reflectivity (albedo) of soil or leaves, respectively
ρ_*vy*_	Albedo of the vineyard (simulated)
τ	Transmittance of the grapevine foliage (for single photons)
ψ_*pd*_	Predawn leaf water potential (MPa)

Based on the allocation of radiation to the vine and soil components, Lebon et al. (2003) formulated equations for potential vine transpiration *T*_0,*v*_ and potential soil evaporation *E*_0_:

(1) $$T_{0,v}=\frac{R_{v}}{R_{vy}}ET_{0},$$

(2) $$E_{0}=\frac{R_{s}}{R_{vy}}ET_{0},$$

where *R*_*v*_, *R*_*s*_, *R*_*vy*_ represent the radiation absorbed by the vines, the soil or the vineyard, respectively, (*R*_*vy*_ = *R*_*v*_ + *R*_*s*_) and *ET*_0_ is the potential evapotranspiration.

We replaced this simple radiation partitioning module (Riou et al., 1989; Lebon et al., 2003) by a numerical simulation approach for three reasons: (1) under conditions of high gap frequency (high porosity) we found that calculated vine transpiration could be substantially higher than measured transpiration; (2) considering the horizontal faces as opaque might overestimate the radiation absorbed by the vines, if the proportion of canopy width to row distance and the porosity are high; and (3) for the use of the model in climate impact studies for entire steep slope grape growing regions, situations described in (1) and (2) are very frequent due to the age of the vineyards (small row distances) and the low SWC (high porosity).

**Figure 2:**
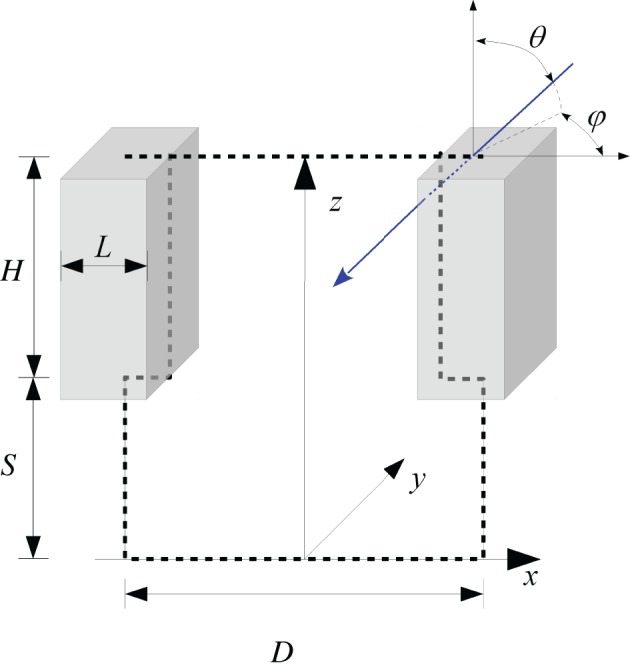
**Outline of the geometrical representation of a row oriented canopy structure**. The blue arrow illustrates the trace of a photon, coming from the direction θ, φ. The travel distance inside the cuboid is shown by the dotted blue line.

We therefore used a numerical simulation approach based on the Monte Carlo method which is widely used in physics to describe radiative transfer (Modest, 2003). We maintained the same geometrical framework in order to keep the input variables unchanged. For a better account for radiation scattered back from soil to the bottom of the foliage we introduced the parameter stem height *S*, representing the distance between foliage and soil surface (Figure 2). The bottom and top side of the canopy were not treated as opaque and the porosity of the canopy was not set to a fixed value as in previous versions (Lebon et al., 2003). Radiative transfer depended on the possible travel distance of radiation inside the cuboid. Radiation extinction in plant canopies is normally modeled by applying the Beer–Lambert law, where the extinction is the product of the extinction coefficient and the cumulated LAI in the pathway (Hirose, 2005). If the leaf area dispersion is assumed to be homogenous, the cumulated LAI can be replaced by the travel distance of radiation inside the cuboid and the extinction coefficient by an expression depending on the porosity of a vertical foliage wall and the corresponding width of the foliage, which were both easy measurable parameters. The model is outlined in more detail in Appendix A.

The model allows us to determine interception fractions for direct and diffuse radiation of the vines and the soil and subsequently to calculate *R*_*v*_, *R*_*s*_, and *R*_*vy*_ in 30 min time steps. This allows a higher frequency of calculations of soil evaporation and vine transpiration as in the original model. Nevertheless, throughout the paper, data are presented for daily time steps based on the sums of each half hour estimation. The corresponding relative fractions of absorbed radiation by grapevines or soil (*f*_*R*,*v*_, *f*_*R*,*s*_) are expressed by:

(3a) $$f_{R,v}=R_{v}/R_{vy},$$

(3b) $$f_{R,s}=R_{s}/R_{vy}.$$

Daily values of *f*_*R*,*v*_ and *f*_*R*,*s*_ were calculated by summarizing *R*_*v*_, *R*_*s*_, and *R*_*vy*_ from radiation data of 30 min temporal resolution.

The interception fractions for direct radiation (grapevines and soil) depend on the direction of the radiation beam relative to the grapevine rows, vineyard slope and aspect and the position of the sun. This adaptation of the model to slopes is described in detail in Appendix B.

The development of height, width, and porosity of the vine canopy are calculated by linear functions depending on thermal time and thresholds for bud burst, hedging, onset and end of leaf abscission as described in detail in Lebon et al. (2003).

### The soil water balance model

Two options exist to adapt a soil water balance model to sloped surfaces. One is to calculate the water fluxes at the normal of the slope surface and the other is to use horizontal equivalents. Since precipitation and soil water content (with vertically installed access tubes) are measured in horizontal equivalents, evapotranspiration is also expressed in l/m^2^ referring to a horizontal surface. All water fluxes or quantities are expressed in l/m^2^ or the equivalent mm.

The soil water balance model is based on the model of Lebon et al. (2003) with some extensions introduced by Celette et al. (2010). The soil water is represented by a reservoir characterized by its total transpirable soil water (*TTSW)*, representing the difference between maximum and minimum (extractable) water content, the transpirable soil water (*TSW*) and the fraction of transpirable soil water (*FTSW* = *TSW*/*TTSW*) remaining at any time during the season (Sinclair and Ludlow, 1986). The reservoir incorporates two sub reservoirs, one for cover crops and one for bare soil. The sub reservoirs are used to calculate individual water balance routines for cover crops and bare soil in order to separate the actual evapotranspiration fluxes between cover crops, bare soil, and grapevines (Celette et al., 2010). Cover Crops can only extract water from the cover crop reservoir, which is therefore characterized by its own *TTSW*_*cc*_. Grapevine roots are present in the complete reservoir and extract water from all sub reservoirs (Celette et al., 2008). Model calculations and data analysis were implemented in the R programming language (R development core team, 2012).

#### Evaporation of bare soil

In the previous model versions (Lebon et al., 2003; Celette et al., 2010) the evaporation of the bare soil was calculated according to Ritchie (1972) and Brisson and Perrier (1991). This part was replaced by the approach of Allen et al. (1998) in the FAO guidelines for computing crop water requirements which has recently been modified to account for small precipitation events and its effects on soil surface evaporation (Allen, 2011). The parameterization of that model seemed more suitable for our application and it has been demonstrated to be robust and apply to different soil types (Allen et al., 1998). Both models (the original one used and the new approach) divide the evaporation process in two stages, where in the first stage evaporation is only limited by the energy available at the soil surface. In the second stage the evaporation rate is lower, because the transport of subsurface water to the evaporating surface is reduced by the dry topsoil layer. That is described by a function depending on the square root of time in Ritchie (1972) and Brisson and Perrier (1991) and by a function depending on the relative content of evaporable water remaining in the evaporation layer in Allen et al. (1998). Allen et al. (1998) assumed that the upper 0.10–0.15 m of the soil layer can be dried by evaporation. This layer is characterized by the total amount of evaporable water (*TEW*) which is the maximum amount of water that can be evaporated during a drying cycle. The amount of water which can evaporate in the first stage is termed readily evaporable water (*REW*) and can be derived from *TEW*. The feedback of the dry topsoil on the evaporation rate in the second stage is described by a soil evaporation reduction coefficient *K*_*r*_ ([0–1], dimensionless) which equals the quotient of the amount of evaporable water actual remaining in the complete evaporation layer to the difference *TEW*–*REW*. To account for small precipitation events, Allen (2011) introduced an additional skin layer to the model, which is located at the topsoil (as a part of the evaporation layer) and its amount of evaporable water is equivalent to *REW*. The skin layer is recharged first by precipitation. In general, water evaporates during the first stage (*K*_*r*_ = 1) if water is available in the skin layer and the reduction of the evaporation rate described by *K*_*r*_ is only effective if the skin layer is dry. Therefore, small amounts of rain falling on a dry soil evaporate more quickly (first stage) as in the previous approach of Allen et al. (1998). The evaporation model of Allen (2011) calculates a daily water balance routine, where *ET*_0_ is one of the input variables. To apply this model to our approach, the water balance routine was calculated for a completely bare soil as described in the dual crop coefficient approach of Allen (2011) (as briefly described above), but instead of the daily *ET*_0_ values the product *f*_*R*,*s*_*ET*_0_ (Equation 3b) is used to account for the potential evapotranspiration effective at the soil surface, which is reduced due to the shading effects of the grapevine canopy. The daily water balance routine calculates a soil evaporation coefficient *K*_*e*_ (depending on *K*_*r*_) with which the evaporation of bare soil of the vineyard *E*_*s*_ can be described by:

(4) $$E=K_{e}f_{R,s}ET_{0},$$

(5) $$E_{s}=E(1-f_{cc}),$$

where *E* is the evaporation of a completely bare soil. The factor *f*_*cc*_ is the area fraction of the soil, which is covered by cover crops and depends on management practices.

The amount of transpiration of grapevines or cover crops from the evaporation layer is neglected in the daily water balance routine as recommended by Allen et al. (1998). *TEW* was estimated from the *TTSW* for the upper 0.15 m soil depth from soil water data of the access tubes and is in line with tabled values of Allen et al. (1998) (Table 3).

**Table 3:** **Total evaporable soil water (*TEW*) and readily evaporable soil water (*REW*) for three experimental vineyards over a soil depth of 0.15 m**.

**Site**	***TEW* (mm)**	***REW* (mm)**
EF	26.9	11.0
BU	21.5	9.2
WI	21.4	9.1

#### Transpiration of grapevines

The approach to calculate the transpiration of grapevines is similar to the model of Lebon et al. (2003). Following Equation (3a), describing the fraction of radiation absorbed by the grapevine canopy, Equation (1) can be rewritten as:

(6) $$T_{0,v}=f_{R,v}ET_{0},$$

where *T*_0,*v*_ is an expression for the potential vine transpiration in the absence of a water deficit. To calculate actual transpiration of grapevines (*T*_*a*,*v*_), *T*_0,*v*_ is multiplied with two coefficients:

(7) $$T_{a,v}=k_{c,v}k_{s}T_{0,v},$$

where *k*_*s*_ is a water stress coefficient [0–1] accounting for the influence of soil water shortage on *T*_*a*,*v*_. The *k*_*s*_ coefficient was introduced by Lebon et al. (2003) to describe the stomatal response to water deficit (Trambouze and Voltz, 2001). This response is described with a bilinear function where during the first stage of water depletion the relative vine transpiration rate, *T*_*a*,*v*_/*T*_0,*v*_ is not limited by available soil water and transpiration is maximal. When *FTSW* falls below a threshold value *p*_*FTSW*_, *T*_*a*,*v*_/*T*_0,*v*_ declines linearly with *FTSW* to zero (Lebon et al., 2003), thus *k*_*s*_ depends on *FTSW* as follows:

(8) $$k_{s}=\{\begin{array}{l} FTSW/p_{FTSW}\;\left(0\leq FTSW\leq p_{FTSW}\right)\\ 1\;\left(p_{FTSW}<FTSW\leq 1\right) \end{array},$$

which is an analogous concept to the framework of *REW* and *TEW* used by Allen et al. (1998) to account for the influence of soil water content on crop transpiration. Since grapevine roots are present in the complete soil water reservoir (Celette et al., 2008), *FTSW* is calculated depending on the total amount of available water over the soil profile, *FTSW* = *TSW*/*TTSW*. The threshold value *p*_*FTSW*_ was set at 0.4 in the previous model based on measurements of stomatal conductance (Lebon et al., 2003). We estimated the threshold value independently using measurements of sap flow and soil water content in this study and found the same value (see Results Sections on sap flow and soil water measurements).

The second factor *k*_*c*,*v*_ is a grapevine specific transpiration coefficient resulting from the sap flow measurements. This factor was necessary to describe the ratio of measured grapevine transpiration (via sap flow) to calculated potential grapevine transpiration (*T*_*a*,*v*_/*T*_0,*v*_) in situations without soil water shortage (*k*_*s*_ = 1). The coefficient *k*_*c*,*v*_ was set to 0.56 as explained in the Results Section. The remaining *TSW*(*i* + 1) of the complete reservoir on any day derives from:

(9) $$TSW(i+1)=TSW(i)+P(i)-ET_{a},$$

where *TSW* is limited to the range 0 ≤ *TSW* ≤ *TTSW*, *i* ∈ {1, 2, 3, …, *n*} refers to the day, *P*(*i*) is the precipitation rate and:

(10) $$ET_{a}=T_{a,v}+ET_{a,cc}+E_{s}$$

is the evapotranspiration of the vineyard, where *ET*_*a*,*cc*_ is the evapotranspiration of the cover crops.

#### Transpiration of cover crops

**Figure 3:**
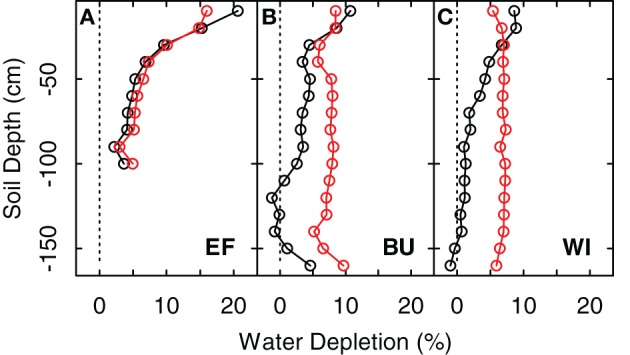
**Extraction profiles and extraction values for three vineyard sites (EF, BU, WI; A–C) expressed as differences between maximum soil water content (*FTSW* = 100%) and soil water content on May 15th 2011 (before grapevine transpiration started) (black line and dots) and the difference between maximum soil water content and soil water content at the end of the growing season in 2011 (1-Nov-2011) (red line and dots)**.

The transpiration rates of the cover crops highly depend on the total transpirable soil water of the cover crop reservoir, *TTSW*_*cc*_, which itself depends on soil characteristics and the soil volume from which the cover crops can extract water. Measurements of extraction profiles of soil water before grapevine transpiration commences in spring showed that soil water was not depleted substantially beyond a depth of 1 m (Figure 3, difference between black line and 0% depletetion), which was therefore used as a good estimate of the root zone of cover crops for all vineyards in this study. This is in line with Celette et al. (2005) who found the same rooting depth for a vine-tall fescue intercropping system in southern France and roughly comparable to values of Allen et al. (1998) for maximum root depth of cool season grass varieties (bluegrass, ryegrass, fescue) of 0.5–1 m. This has also been confirmed by direct measurements on different species in the region of the present study (Uliarte et al., 2013). However, Celette et al. (2008) observed a maximum depth of soil water use by cover crops of 1.5 m under very dry conditions in the south of France. Based on our measurements and the assumption that a relatively higher frequency of summer rainfall at the study sites may prevent the necessity of cover crop plants to exploit soil depths beyond 1 m, we calculated the *TTSW*_*cc*_ of the cover crop reservoir as:

(11) $$TTSW_{cc}=TTSW(1\;m)f_{cc},$$

where *TTSW*(1 m) refers to the *TTSW* of the upper 1 m soil layer and *f*_*cc*_ represents the area fraction covered by cover crop plants (Equation 5).

The daily remaining water in the cover crop reservoir is therefore computed by:

(12) $$\begin{array}{l} TSW_{cc}(i+1)=TSW_{cc}(i)+P(i)f_{cc}-ET_{a,cc}(i)\\ \;-k_{s,v,cc}k_{c,v}T_{0}, \end{array}$$

where *TSW*_*cc*_(*i* + 1) is the remaining transpirable soil water kept within the range of 0 ≤ *TSW*_*cc*_ ≤ *TTSW*_*cc*_ and *P* is the precipitation. The last term in Equation (12) is the amount of water extracted by the grapevines from the cover crop reservoir. Based on the condition that the sum of extracted water from the sub reservoirs must equal the actual grapevine transpiration:

(13) $$T_{a,v}=k_{s}k_{c,v}T_{0,v}=\left(k_{s,v,cc}+k_{s,v,r}\right)k_{c,v}T_{0,v},$$

where *k*_*s*,*v*,*cc*_ and *k*_*s*,*v*,*r*_ are coefficients integrating the partitioning of transpiration and the feedback of water stress appearing in the cover crop (*k*_*s*,*v*,*cc*_) or the remaining (non-cover crop, *k*_*s*,*v*,*r*_) reservoir onto grapevine transpiration. A case differentiation considering the feedback of water stress from the complete or the individual sub reservoirs results in:

(14) $$\begin{array}{l} k_{s,v,cc}=\\ \;\{\begin{array}{ll} \frac{TSW_{cc}}{p_{FTSW}TTSW} & \left(k_{s}<1\right)\\ \frac{TTSW_{cc}}{TTSW} & \left(k_{s}=1\wedge \frac{TSW_{cc}}{TTSW_{cc}}\geq p_{FTSW}\wedge \frac{TSW_{r}}{TTSW_{r}}\geq p_{FTSW}\right)\\ \frac{TSW_{cc}}{p_{FTSW}TTSW} & \left(k_{s}=1\wedge \frac{TSW_{cc}}{TTSW_{cc}}<p_{FTSW}\wedge \frac{TSW_{r}}{TTSW_{r}}\geq p_{FTSW}\right)\\ 1-\frac{TSW_{v}}{p_{FTSW}TTSW} & \left(k_{s}=1\wedge \frac{TSW_{cc}}{TTSW_{cc}}\geq p_{FTSW}\wedge \frac{TSW_{r}}{TTSW_{r}}<p_{FTSW}\right) \end{array}, \end{array}$$

where *TSW*_*r*_ and *TTSW*_*r*_ are the transpirable or the total transpirable soil water of the remaining reservoir (*TSW*_*r*_ = *TSW* − *TSW*_*cc*_, *TTSW*_*r*_ = *TTSW* − *TTSW*_*cc*_), respectively.

In order to estimate the contribution of cover crops to water use throughout the annual cycle we followed the system devised by Allen et al. (1998). They divided the growing season into four growth stages, an initial stage, a development stage, a mid-season stage, and a late-season stage. The start of the cover crop growing season was set at 7 days before the last occurrence of −4°C (air temperature) in spring (usually beginning of March but can be substantially earlier) and the end at 7 days after the first −4°C in fall/winter (usually end of November – beginning of December) (Allen et al., 1998). The start denotes the onset of the initial stage and the end date denotes the start of the late season stage. To avoid that a late spring frost occurring in April or May would artificially retard cover crop development (because of the 7 day before −4°C rule), events such as these are ignored in the current model. As evapotranspiration coefficients were not available for the native vegetation at the experimental sites, it was assumed that the cover crops are not active during the late and initial stages and that evapotranspiration only occurs as evaporation (Allen et al., 1998). This has recently been confirmed by direct measurements (Uliarte et al., 2013). During the development period cover crops grow and reach full ground cover at the end of this stage so that evapotranspiration during the mid-season follows actual evapotranspiration of the crops. The transition from evaporation to evapotranspiration was described by a ground cover coefficient *f*_*g*_, which equals 0 during the late and initial stage, increases linearly from 0 to 1 during the developmental stage and equals 1 during the mid-season stage. The duration of the initial and development stage was set to 30 and 50 days, respectively, which provided good results in spring and is in agreement with local observations (Uliarte et al., 2013). This process could clearly be refined if a degree day system would be used or other plant growth models.

The evapotranspiration of cover crops (*ET*_*a*,*cc*_) is then calculated as:

(15) $$ET_{a,cc}=f_{cc}\left(f_{g}k_{s,cc}f_{R,s}ET_{0}+\left(1-f_{g}\right)E\right),$$

where *k*_*s*,*cc*_ is the cover crop water stress coefficient [0–1], calculated in analogy to Equation (8) for *FTSW*_*cc*_ = *TSW*_*cc*_/*TTSW*_*cc*_ and a threshold value for *p*_*FTSW*_ = 0.4 as reported for rye grass in Allen et al. (1998).

### Weather data, surface runoff and evapotranspiration

Weather data were provided by weather stations of the Geisenheim branch office of the Deutscher Wetterdienst (Germany's National Meteorological Service, DWD). The climate in Geisenheim can be categorized as humid temperate. Annual precipitation is 544 mm (1981–2010) (DWD) and is approximately equally distributed throughout the year (maximum in July with 60 mm, minimum in April with 35 mm). Light precipitation events (<10 mm/day) dominate and contribute 65% of total precipitation, whereas daily precipitation events larger than 20 mm contribute only 9%, respectively. Severe precipitation events are rare, the three highest amounts of daily rainfall ever recorded (1981–2010) were 75 mm (6-Jul-1999), 52 mm (13-Aug-1995), and 37 mm (9-Aug-1981). Emde (1992) showed that under these circumstances no surface runoff occurs if cover crops are used. He also demonstrated that surface runoff depended on precipitation intensities on very short time scales (minutes) and that clean cultivated vineyards soils were most vulnerable. We therefore assumed that the total amount of surface runoff was generally negligible and only rainfall amounts exceeding soil storage capacity were treated as lost, whereby no distinction was made between losses as surface runoff or deep percolation. Mean *ET*_0_ between April 1 and September 30 is 605 mm.

For EF and BU, weather data of a station located directly in the EF plot were used which provided temperature, wind speed, precipitation, relative humidity and global solar radiation. For WI the same data with the exception of solar radiation was available from a nearby weather station (<200 m distance). For this site, radiation data from the main station at Geisenheim (3 km distance to WI) were used which also provided the direct and diffuse fractions of global radiation.

In order to estimate these components for EF and BU, a correlation between the diffuse fraction of global radiation and a clearness index as described in Duffie and Beckman (2006) was derived from the Geisenheim data and assumed to be valid for the EF and BU sites. The correlation is outlined in Appendix C. The extraterrestrial radiation of the steep slope sites (needed to calculate the clearness index) was calculated as described by Allen et al. (2006).

Potential evapotranspiration was calculated according to Allen et al. (2005) taking into consideration that net radiation at the slope surface is altered. We therefore projected the solar radiation from the horizontal to the slopes by using the HDKR model (Reindl et al., 1990; Duffie and Beckman, 2006) with radiation partitioning (diffuse-direct) calculated by Equations (C1, C2). Longwave radiation emitted or reflected from the surrounding topography was neglected because a simple estimation based on the assumption that the slope emits as much longwave radiation to the surrounding terrain (assumed to be horizontal) as it receives, so that only the net longwave radiation part related to the view factor of the slope to the sky is considered, increased the potential evapotranspiration for EF (35° slope) by only 1%. The resulting potential evapotranspiration refers to the surface of the slope (*ET*_0*s*_) and was re-projected to the horizontal to calculate the horizontal equivalent of evapotranspiration (to be congruent with precipitation data in the water balance calculation, Allen et al., 2006) by:

(16) $$ET_{0}=ET_{0s}/\cos\beta$$

where β is the slope angle.

### Climate change risk analysis

Projections of possible future water budget changes for the three vineyard sites were calculated by feeding the described water budget model with the data of a small ensemble of four Regional Climate Models (RCMs). The used RCMs were different in their downscaling approaches (statistic or dynamic) and/or in the GCM [ECHAM5/OM, Max-Planck-Institute of Meteorology (MPI-M) in Hamburg, Germany or HadCM3, Met Office Hadley Center in Exeter, UK] driving them. All projections were for the A1B emission scenario of the IPCC (Nakicenovic et al., 2000). The climate projections used were: (1) A projection for the Geisenheim weather station of the statistical model WETTREG2010 (Kreienkamp et al., 2010) driven by ECHAM5/OM, (2) two projections of the dynamic RCM CLM model (Rockel et al., 2008), one driven by ECHAM5/OM (Lautenschlager et al., 2009) and one driven by HadCM3 (Schär and Christensen, 2013), and (3) one projection of the dynamic RCM REMO/ECHAM5 (Jacob, 2005). Additionally, original daily weather data from 1955 to 2012 of the weather station in Geisenheim were available.

The grid box data of the dynamic RCMs are areal average values and cannot reproduce the variability of small scale precipitation, which is high around Geisenheim because of the local orography. In general, modelers recommend to aggregate over several grid boxes and to finally perform a spatial averaging of the results of the impact model (Kreienkamp et al., 2012). The impact model in this study needs site-specific data, a spatial averaging of the results is therefore not reasonable. To overcome this discrepancy between the spatial scale of the RCM data and the site-specific character of the study (Maraun et al., 2010), the time series of 9 grid boxes covering the area of the experimental site (one enclosing the plots and eight around) were evaluated. The comparison of the 9 time series per model revealed that they differed in the calculated absolute numbers of drought stress days (mainly caused by the different bias of mean annual precipitation compared to the observed data), but showed very similar temporal courses and change signals. Therefore, only the results of the grid box are shown which revealed the smallest difference between original and calculated number of drought stress days for the period from 1971 to 2000.

The results were meant to form the basis for a site-specific evaluation with respect to possibly increasing risks of developing a higher frequency of drought events. The evaluation of drought stress occurrence and severity is based on a relationship between *FTSW* and vine predawn leaf water potential (ψ_pd_) reported by several authors (Lebon et al., 2003; Pellegrino et al., 2004; Gruber and Schultz, 2005; Schultz and Lebon, 2005). Since ψ_pd_ is a widely used physiological parameter to quantify plant water deficit and since it has been related to many physiological responses in the vegetative and reproductive development of plants (Williams and Matthews, 1990) it provides the opportunity to couple soil and plant water status for the estimation of future developments. The relationship *FTSW* to ψ_pd_ has proven to be valid over a large scale of different soil water holding capacities and for different vineyard sites (Gruber and Schultz, 2005). From the published data of these authors follows, that the common threshold value for severe stress of ψ_pd_ = −0.6 MPa corresponds with *FTSW* values in the range of 0 ≤ *FTSW* ≤ 0.2. Since it is uncertain if the water balance model can account correctly for small changes in that extreme dry range and because of the limited amount of data available from field experiments, the threshold for severe water stress was set to *FTSW* ≤ 0.15. With this threshold it was possible to classify the water availability of each day with respect to its physiological consequences and to sum up the number of days in the range of severe water stress over the growing season (1 May–30 September).

### Measurements to validate the water balance model

#### Soil and plant water status measurements

Soil water status measurements were performed with a portable capacitance sensor system (Diviner 2000, Sentek, Australia) based on the frequency domain reflectometry technique. Because of differences of soil texture between sites or soil depth, the default calibration equation of the manufacturer was used to estimate soil water content. Following the *FTSW* concept (Section The Soil Water Balance Model), soil water content was therefore expressed as differences (*TTSW*, *TSW*) or relative changes (*FTSW*). In each vineyard we installed at least six soil water access tubes up to a depth of maximal 1.60 m. The tubes were positioned vertically (not in the normal of the sloped surface) and thus measured horizontal equivalents of soil water content. In EF and BU not all tubes reached this depth because of thin soil layers above the bedrock at these sites. The *TTSW* of EF and BU was estimated from the difference between maximum and minimum water content over several seasons and the entire soil/root profile (Sinclair and Ludlow, 1986). For WI, the *TTSW* was estimated from measurements of vine predawn leaf water potential (ψ_pd_) and the established relationship between *FTSW* and ψ_pd_ previously reported (Lebon et al., 2003; Gruber and Schultz, 2005), because a minimum water content was not reached during the study period. There are also some doubts with respect to covering the entire rooting depth with a measurement technique which is limited to a depth of 1.60 m. However, this certainly covers the main water extraction reservoir of vineyard soils.

Water potential at WI was determined with a pressure chamber predawn (Soilmoisture Corp. Santa Barbara USA) on six fully expanded leaves per treatment and date.

Soil water measurements were performed in weekly time steps except during the winter months where 2–4 week intervals were chosen to monitor the refilling of the soil and to find the maximum point of replenishment. Two access tubes, equipped with a permanent measuring technique (Enviroscan, Sentek, Australia) additionally monitored the soil water content at five measuring depths in EF to have more information with a higher temporal resolution.

#### Sap flow measurements

Sap flow was measured from June until the end of the growing season on six grapevines in each vineyard with custom made Granier-type sap flow sensors. This measurement technique has been adopted to grapevines and validated by Braun and Schmid (1999b). Trunk cross sections were roughly elliptical shaped, the length of the mean minor and major axis were 22/27 mm (EF), 33/41 mm (BU), and 33/39 mm (WI). We used probes with a length of 18 mm for BU and WI, and 14 mm for EF. The probes were inserted into the trunk between 10 cm above the graft union and 10 cm below the pruning zone with a distance between the probes of approximately 15 cm on trunk segments which were free of wounds. The segments were insulated with foam material and aluminum foil in the area of the installed probes. The constant heating power was adjusted to 0.20 W for BU and WI and to 0.16 W for EF, to ensure a constant heat output per unit probe length in the range of previously reported applications (Lu et al., 2004). The original calibration equation of Granier (1985) was used as Braun and Schmid (1999b) found this equation to be valid for grapevines over a wide range of sap flux densities. Nocturnal sap flow was not considered, because an analysis of potential evapotranspiration on 30-min temporal resolution showed that the occurrence of a substantial evaporative demand of the atmosphere during nights were rare events for the climate conditions of the study area.

#### Porosity measurements

The porosity of the canopy is an important parameter for the estimation of the distribution of radiation within the canopy and consequently for the estimation of canopy water use. We therefore estimated canopy porosity of the experimental sites every year a few weeks before harvest by taking digital *RGB* pictures of the vine stocks with fully developed foliage (width 40–45 cm, perpendicular to the vertical foliage walls) which were used for sap flow measurements.

*RGB* pictures were also used to validate Equation (A4), which describes the relationship between the porosity and the travel distance of the radiation in the foliage. Therefore, pictures of a square of 70 cm (serving as an image detail of a vertical foliage wall) were taken from a distance of 5 m at different viewing angles along a horizontal semicircle resulting in different distances the light had to travel across the foliage.

A white sheet was always used to provide a background behind the vine row. We classified each pixel of the pictures by using chromatic coordinates (Sonnentag et al., 2012) and appropriate thresholds assessed by kernel density estimation and were able to calculate the porosity values. The R package biOps (Bordese and Alini, 2012) was used for image processing.

## Results

### Radiation partitioning

**Figure 4:**
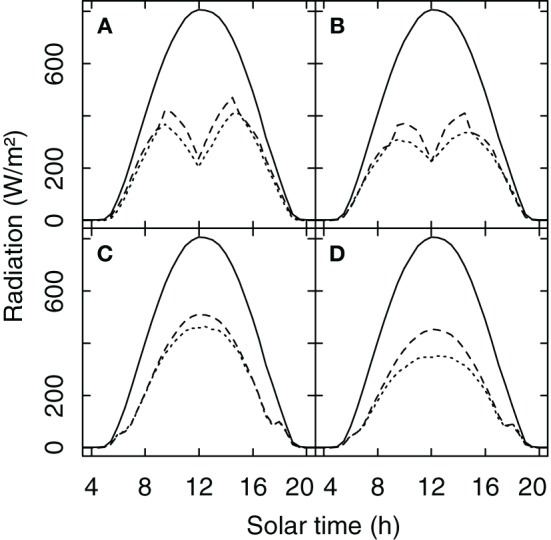
**Global radiation and the simulated amount of radiation received by a row oriented grapevine canopy (*R*_*v*_) (row distance 1.60 m, width = 0.40 m, height 1.10 m, 0.80 m above ground)**. For North-South **(A,B)** and for South-East **(C,D)** row orientation and for porosity levels of the foliage of 0.25 **(A,C)** and 0.5 **(B,D)**. Solid lines show global radiation for a clear sky day in Geisenheim, Germany (20-Aug-2011), the dashed lines show *R*_*v*_ simulated with the model of Riou et al. (1989), and the dotted line *R*_*v*_ calculated with a Monte Carlo simulation.

A comparison between the original radiation model of Riou et al. (1989) and the new Monte Carlo approach showed very similar results for the amount of radiation received by the grapevine canopy for a porosity level of 0.25 which would be indicative of average to vigorous growing conditions (Figures 4A,C). For situations with lower vigor (porosity = 0.5) the simulated *R*_*v*_ of the Riou et al. (1989) model is higher than the Monte Carlo simulation (Figures 4B,D). That is likely due to the fact that the Riou et al. (1989) model treats the horizontal faces as opaque which artificially increases radiation absorption specifically at small ratios of row distance to canopy width.

### Measurement of porosity for different light travel distances inside the foliage

**Figure 5:**
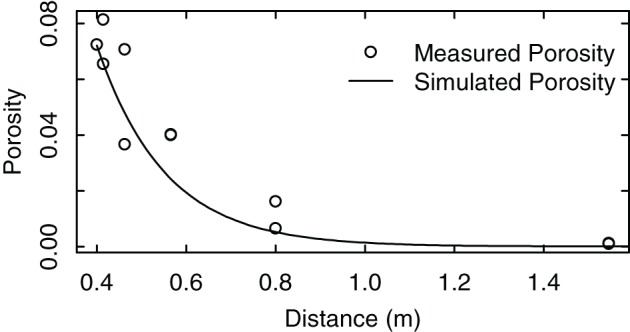
**Measured porosity of a grapevine row trained to a VSP trellis. The distance equals the length a solar beam has to travel to traverse the foliage**. The measurements were taken from RGB pictures of a vertical square of 0.70 m positioned in front of a vertical foliage wall. Pictures were taken from a distance of 5 m at different viewing angles along a horizontal semicircle.

We compared the measured porosity values with the calculated values in their dependence on light travel distances within the canopy (Equation A4, Figure 5). The results showed that the decrease of porosity with the increase of light travel distance could be well approximated by Equation (A4). Refinements of this approach may have to take into account measured leaf area distributions or inhomogeneous leaf angle dispersion inside the canopy due to different canopy forms and shoot orientation.

### Site characteristics

**Figure 6:**
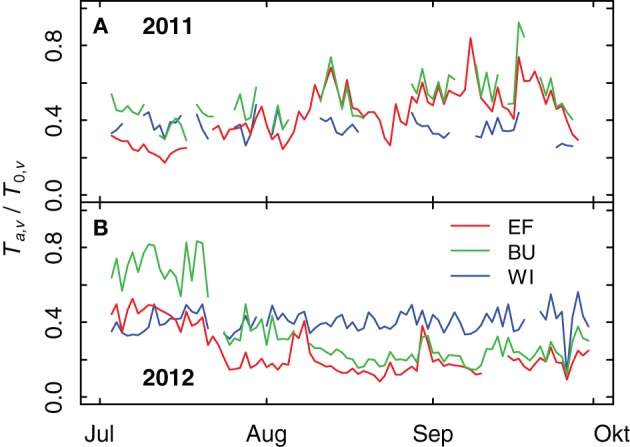
**Relative grapevine transpiration (*T*_*a*,*v*_/*T*_0,*v*_) for three vineyards (EF, BU, WI) with different soil water holding capacities during 2011 (A) and 2012 (B)**. Actual grapevine transpiration was measured by sap flow, potential transpiration was calculated from weather data and modeled vineyard characteristics.

Differences in *TTSW* of the plots caused differences in grapevine transpiration rates as measured by sap flow. These differences (expressed as relative transpiration, *T*_*a*,*v*_/*T*_0,*v*_) were more pronounced in 2012 than in 2011 (Figure 6). In 2011 the ratio of *T*_*a*,*v*_/*T*_0,*v*_ of EF (smallest *TTSW*) was significantly lower (tested with a pair-wise comparison (*p* < 0.1) of an analysis of variance of the relative transpiration rates of 6 vines per vineyard for each day) in the first half of July compared to BU and WI, which can be explained by a short period with low rainfall at the end of June and a decrease in soil water content (see Figures 9A,D). High rainfall amounts during August and September 2011 resulted in an increase of soil water content in all vineyards (see Figure 9) and in an increase of relative transpiration rates for EF and BU (Figure 6A). During that period the mean values of *T*_*a*,*v*_/*T*_0,*v*_ for WI were lower compared to EF and BU, but significant differences appeared only on a few days. In 2012 the ratio of *T*_*a*,*v*_/*T*_0,*v*_ was highest for BU during the first half of July (significant, Figure 6B). Thereafter, *T*_*a*,*v*_/*T*_0,*v*_ declined first in EF (smallest *TTSW*), followed by BU, probably caused by a decrease in soil water content (cf. Figure 9). In WI (high *TTSW*) the ratio remained almost constant during both growing seasons and was significantly higher than EF and BU during August and September 2012, except for brief recoveries of transpiration rates in EF and BU caused by intermittent precipitation events (Figure 6B).

### Evaluation of model parameters

#### Grapevine transpiration coefficient *k*_*c*,*v*_

The transpiration coefficient *k*_*c*,*v*_ in Equation (7) represents the ratio of *T*_*a*,*v*_/*T*_0,*v*_ under conditions where soil water content does not limit transpiration (i.e., *k*_*s*_ = 1). It had to be introduced because measured transpiration rates (sap flow) never matched calculated potential transpiration rates despite the fact that previous versions of the model adequately described soil water content dynamics (Lebon et al., 2003; Pellegrino et al., 2004), yet individual components [vine transpiration and soil (+cover crop)] had never been individually validated. We therefore determined the *k*_*c*,*v*_ value for each of the three vineyards by calculating the mean of the daily ratios of measured sap flow (*T*_*a*,*v*_) to calculated potential grapevine transpiration (*T*_0,*v*_) for periods where drought stress was absent (*FTSW* > 0.4). The *k*_*c*,*v*_ value then used in the model represented the mean of the individual *k*_*c*,*v*_ values (Table 4).

**Table 4:** **Ratios of actual to potential transpiration of grapevines for three different vineyards during periods well supplied with water**.

**Vineyard**	****k*_*c*,*v*_***
EF	0.57 ± 0.14
BU	0.68 ± 0.32
WI	0.42 ± 0.22
Mean	0.56 ± 0.32

*Error values represent means of the confidence intervals (p < 0.05) of daily sap flow data of six vines per vineyard*.

#### Influence of soil water availability on grapevine transpiration

Sap flow and soil water content data were used to validate if the bilinear function of Equation (8) is capable to describe the dependence of transpiration, as *T*_*a*,*v*_/*T*_0,*v*_, on soil water availability (*FTSW*) and to assess if the selected threshold value *p*_*FTSW*_ = 0.4 (Lebon et al., 2003), which differentiates non-water limiting and water limiting stages accurately reflects the situations at our experimental sites.

**Figure 7:**
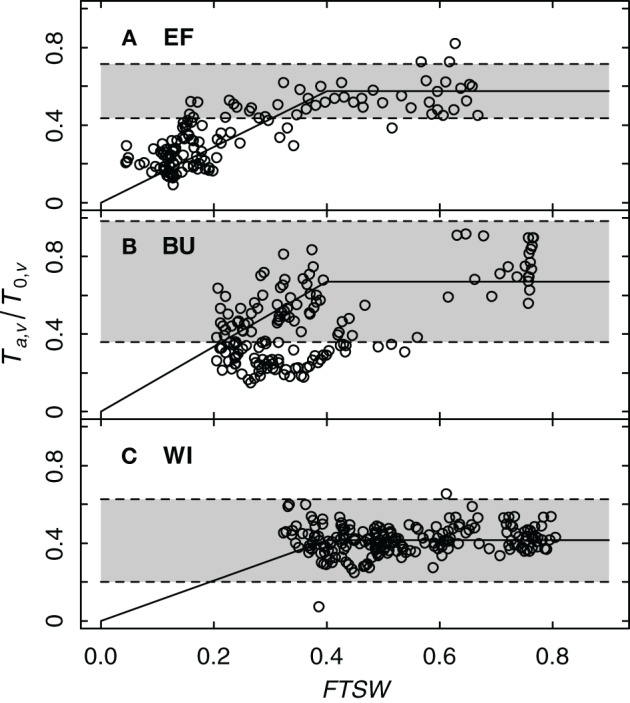
**Relative grapevine transpiration (*T*_*a*,*v*_/*T*_0,*v*_) as a function of *FTSW* for three vineyards (EF, BU, WI; A–C)**. The shaded background indicates the confidence interval of *T*_*a*,*v*_/*T*_0,*v*_, where transpiration is not limited by insufficient water supply (resulting from sap flow data of six vines per vineyard for the years 2011 and 2012). The solid line shows a bilinear function describing the feedback of water stress on relative grapevine transpiration with a threshold value of 0.4.

To get more data points on *FTSW*, which was determined weekly, data for each day were estimated by linear approximation between successive measurements. Figure 7 shows the ratio of *T*_*a*,*v*_/*T*_0,*v*_ as a function of *FTSW* for the three experimental vineyards. Following Equation (7) the ratio of *T*_*a*,*v*_/*T*_0,*v*_ equals the product of the transpiration coefficient *k*_*c*,*v*_ and the water stress coefficient *k*_*s*_. Since the transpiration coefficient is constant (Table 4), a deviation from this value indicates the onset of water deficit caused by a decrease of *k*_*s*_ to values < 1. The *FTSW* value at which this happens denotes the threshold *p*_*FTSW*_. This value was estimated in our case from *FTSW* values where *T*_*a*,*v*_/*T*_0,*v*_ data decreased below the lower limit of the confidence interval of *k*_*c*,*v*_ (Table 4) suggesting the onset of water deficit. Figure 7 shows, that for all three vineyard sites a value of *p*_*FTSW*_ = 0.4 described reasonably well the point at which this deviation occurred confirming the Lebon et al. (2003) approach (determined by measurements of stomatal conductance) with sap flow data.

#### Influence of environmental conditions on grapevine transpiration

**Figure 8:**
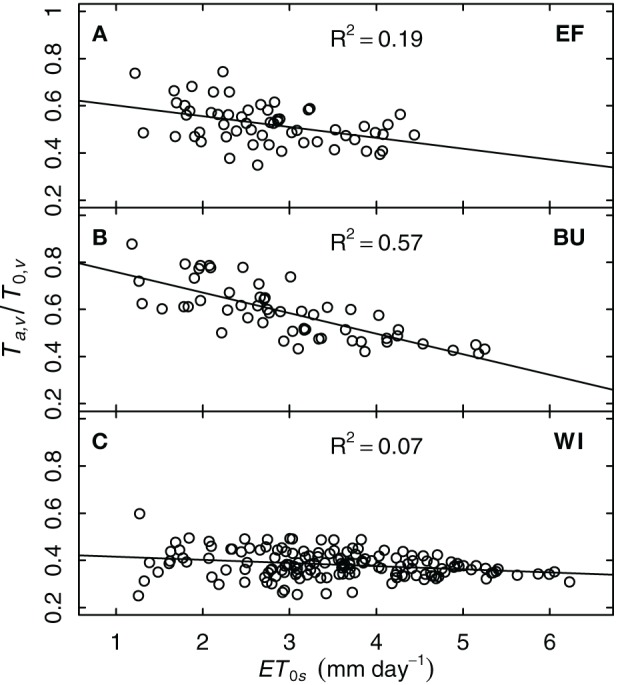
**Relative grapevine transpiration (*T*_*a*,*v*_/*T*_0,*v*_) for three vineyards (EF, BU, WI; A–C) as a function of potential evapotranspiration at the vineyard surface (*ET*_0*s*_, including the slope) for situations without water stress**. *T*_*a*,*v*_ was based on sap flow data of six vines per vineyard from the years 2011 and 2012.

Stomatal conductance of grapevines can be sensitive to vapor pressure deficit (*VPD*). Since *T*_*a*,*v*_/*T*_0,*v*_ can also be taken as an indicator of whole-plant conductivity for water, one could expect a decrease of *T*_*a*,*v*_/*T*_0,*v*_ with increasing *VPD*. Since *VPD* effects have a diurnal pattern and model and measurements were on daily time-steps, we investigated the relationship between *T*_*a*,*v*_/*T*_0,*v*_ and *ET*_0_, whereby *ET*_0_ integrates more environmental variables to express the evaporative demand the plants are exposed to. To exclude the influence of soil water shortage, only data during periods without drought stress (*FTSW* > 0.4, *k*_*s*_ = 1) were examined. The strongest correlation was found between *T*_*a*,*v*_/*T*_0,*v*_ and *ET*_0*s*_ [i.e., for BU: *R*^2^ = 0.57, *T*_*a*,*v*_/*T*_0,*v*_ = *f*(*ET*_0*s*_)] but this was not consistent for all plots (Figure 8). Only the steep slope sites EF and BU showed a decrease in relative transpiration rate with increasing evaporative demand, whereas WI exhibited only a small response.

### Validation of the water balance model

#### Simulations of the soil water budget

**Figure 9:**
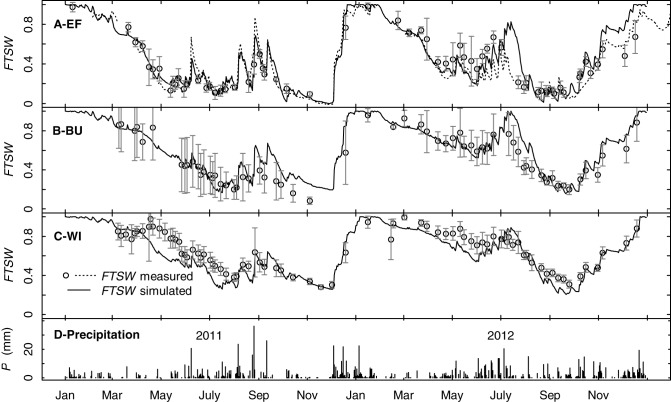
**Simulated and measured seasonal time courses for precipitation (D) and the fraction of transpirable soil water (*FTSW*) over the rooting profile for three vineyards (EF, BU, WI) and the years 2011 and 2012 (A–C)**. Solid lines represent simulated *FTSW*, symbols represent measured *FTSW* (means of at least 5 access tubes per vineyard, error bars refer to confidence intervals, *p* < 0.05). The dotted line in **(A)** represents one continuously measuring access tube in EF.

Simulations with the water budget model over two years showed that the model traced measured *FTSW* values of the three vineyards well and was able to mimic the dynamics in soil water content during different seasons including soil recharge in winter and the transition from evaporation to evapotranspiration due to cover crop development in spring (Figures 9A–C). Changes in *FTSW* in EF in summer resulted mainly from changes in the upper soil layer (0–30 cm, data not shown). Precipitation caused more rapid responses of *FTSW* in EF compared to BU and WI because of the lower *TTSW*. This can also be seen from the course of daily data of one continuous measuring tube in EF (Figure 9A). *FTSW* in WI was slightly underestimated in both years in spring. In general the model was able to operate on small time scales and was capable to cover the effects of canopy development and different management practices on whole vineyard water consumption.

#### Simulation of grapevine transpiration

**Figure 10:**
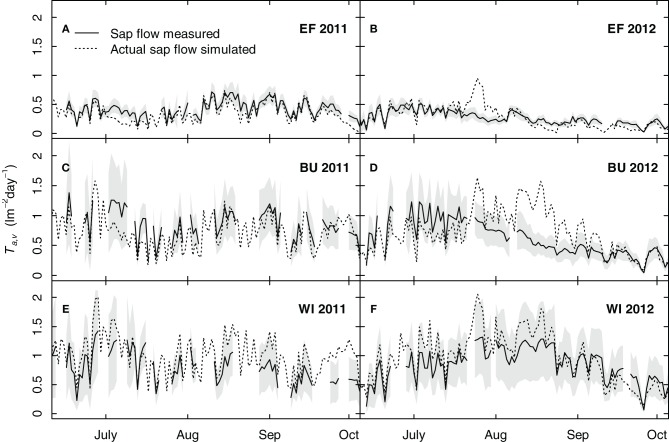
**Simulated and measured seasonal courses of grapevine transpiration for three vineyards (EF, BU, WI) and 2 years [2011 (A, C, E), 2012 (B, D, F)]**. Solid lines show measured transpiration rates by sap flow (means of 6 vines per vineyard), the shaded area shows the confidence interval (*p* < 0.05) and dotted lines show the simulated transpiration rates (calculated with an uniform grapevine transpiration coefficient).

Figure 10 shows simulated grapevine transpiration rates using a uniform transpiration coefficient (*k*_*c*,*v*_ = 0.56, Table 4). A comparison between measured and simulated sap flow showed that the model could reproduce sap flow within the measured confidence intervals for most parts of the seasons, sites and years. A distinct overestimation was calculated for BU in 2012 (Figure 10D). This overestimation between mid July and the end of August was 18 mm as compared to the measured mean values, yet it was not reflected in the soil water budget (Figure 9B). The nearly consistent and small overestimation of transpiration for WI (Figures 10E,F) was related to the used uniform grapevine transpiration coefficient in the simulations, which was slightly higher than the site specific one. In contrast to EF and BU no impact of soil water shortage on grapevine transpiration was detectable for WI (Figures 10E,F) over both growing seasons.

#### Sensitivity analysis

Sensitivity analyses of previous versions of the model or parts thereof were already conducted for several parameters (Trambouze and Voltz, 2001; Lebon et al., 2003; Celette et al., 2010). Two new model aspects were analyzed here. First, the adaptation to steep slopes was evaluated to quantify the impact of the degree of slope and slope orientation on potential evapotranspiration and, second, the introduction of the grapevine transpiration coefficient *k*_*c*,*v*_ was assessed for its impact on water use.

Annual *ET*_0_ increased by about 25% between an inclination angle of 0°–30° with a south orientation (Table 5) indicating that sloped areas face a substantially higher risk of developing water deficit independent of soil type and depth. This effect has two reasons, one is that the surface receives more solar energy to evaporate water and the second is that the evaporating surface per horizontal equivalent increases.

**Table 5:** **Calculation of annual sums of the horizontal equivalent of potential evapotranspiration, *ET*_0_, for a slope (50° latitude North, Geisenheim) with different inclination angles (in ° and % slope) and aspects using weather data of 2012 (Geisenheim weather station, DWD)**.

		***ET*_0_ (mm/year)**				
**Aspect**		**S**	**SW/SE**	**W/E**	**NW/NE**	**N**
**Inclination**						
0° (0%)		800	800	800	800	800
5° (9%)		823	818	802	786	779
10° (17%)		850	840	811	777	762
15° (25%)		882	868	825	771	748
20° (33%)		919	902	846	771	737
25° (41%)		961	942	874	776	731
30° (48%)		1012	991	910	789	729

The introduced grapevine transpiration coefficient, *k*_*c*,*v*_, was set to 0.56 as a result of experimental data from the three vineyard sites. Consequently the model calculated only about half of the grapevine transpiration rates compared to the approaches of Lebon et al. (2003) and Celette et al. (2010) who did not use a coefficient and did not try to validate grapevine transpiration against an independent measurement method such as sap flow. Running the model with a *k*_*c*,*v*_ value of 1 led to an underestimation of soil water content. The simulated mean *FTSW* (May–September) was reduced by 22 and 20% for EF, 32 and 28% for BU, and by 29% for WI for the years 2011 and 2012, respectively. Thus, the underestimation increased with increasing ratio of grapevine transpiration to actual evapotranspiration. This ratio is low in EF because of wide row spacing and reduced grapevine transpiration rates as a consequence of frequent water shortage, but high in WI, where grapevines did not suffer water shortage. Compared to the large differences caused by different *k*_*c*,*v*_ values, the effect of deviations of calculated to measured grapevine transpiration rates (Figure 10) on vineyard soil water content was low (see Figure 9). This is probably related to the interactions between vine and cover crop water use, respectively, soil evaporation which had compensatory effects on the development of *FTSW*.

### Comparison of different simulated evapotranspiration fluxes

**Figure 11:**
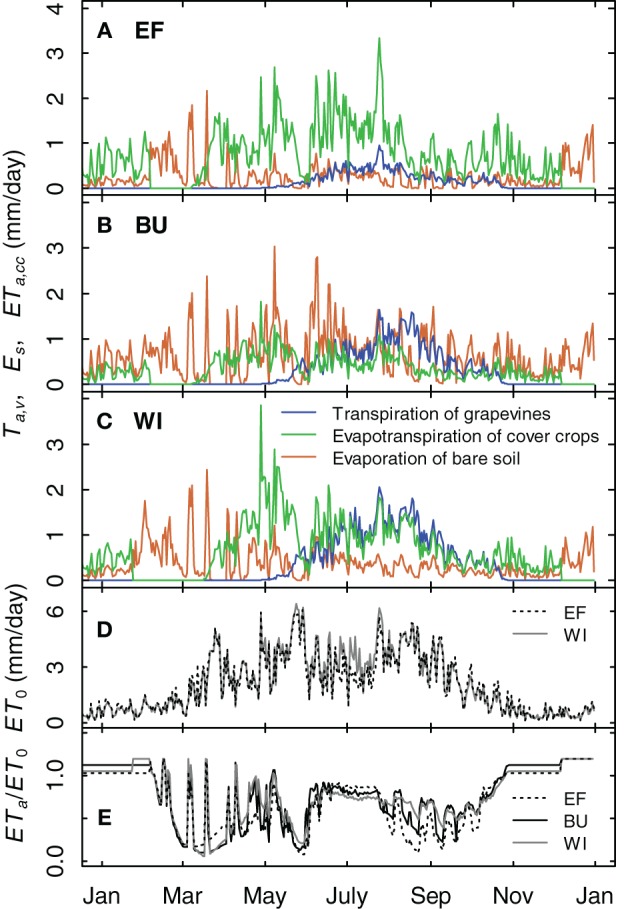
**Simulation examples for the transpiration of grapevines, the evapotranspiration of cover crops and the evaporation of bare soil for three vineyards for 2012 (A–C)**. The cover crop development is divided into growing stages. It is assumed that the cover crops are destroyed by severe frost and recover during spring. **(D)** Potential evapotranspiration for site EF and WI. **(E)** Simulated relative evapotranspiration of the vineyards.

A comparison of the different simulated water fluxes of the vineyards for the year 2012 showed the effects of different row distances (Table 1) and soil management practices (inter-rows with cover crops in EF and WI, alternating bare soil and cover crop in BU, Table 1) on soil water budget (Figure 11). The fraction of grapevine water consumption of the vineyards actual evapotranspiration was 18% for EF (2.50 m row distance) and 38% and 45% for BU und WI (1.60 m row distance), respectively, during the period with fully developed canopy. Relative evapotranspiration (expressed as *ET*_*a*_/*ET*_0_) was maximum during the winter months (Figure 11E) due to wet soil and humid weather, but declined rapidly in late winter/early spring in all plots (Figures 11A–C,E), during the transition of mainly cover crop to mainly bare soil and back. Absolute values for the evapotranspiration of cover crops in WI were in the range of 1–4 mm/day after recovery in spring and between 1 and 2 mm/day from June to the end of August associated with a developed grapevine canopy and high *ET*_0_ values. Values for EF were slightly higher during that period (0.5–3 mm/day), because of the wider row spacing. Evaporation from bare soil was over the year the most dominant water loss process for BU. Only when grapevines had developed a full canopy, transpiration did exceed soil evaporation.

### Assessment of climate change impact on future vineyard water budget

**Figure 12:**
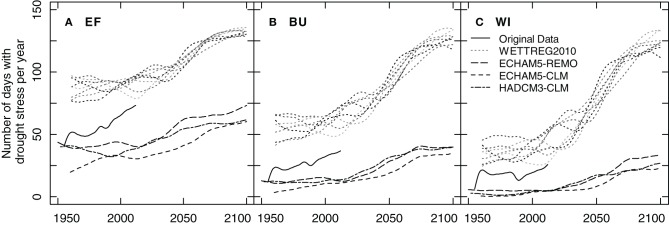
**Smoothed 30-year running means of the yearly sum of days with drought stress for three different vineyards (EF, BU, WI; A–C), calculated using a soil water balance model, original weather data (from Geisenheim) and projections of regional climate models**. Drought days were defined as days when predawn water potential decreased to −0.6 MPa.

Model runs with original (1955–2012) and climate projection data incorporating specific site characteristics were performed for EF, BU, and WI. The analyses revealed that the number of days with drought stress (*FTSW* < 0.15; closely equivalent to ψ_pd_ = −0.6 MPa) between 1 May and 30 Sep. (152 days) has already increased significantly (*p* < 0.05; Mann–Kendall trend test; McLeod, 2011) in the past for the sites EF and BU but not for WI (Figure 12). For WI 64% of the years had almost no days with drought stress and a substantial number of stress days (>20 days) occurred during 26% of the years (Figure 12C).

Climate models differed substantially in their projections of absolute numbers of drought occurrence both for the past and for the future. As a result of model specific biases compared to the Geisenheim weather station, ECHAM5-REMO, ECHAM5-CLM, and HADCM3-CLM underestimated and WETTREG2010 overestimated the frequency of drought days for the past (Figures 12A–C). The strongest increase in the number of drought stress days was projected by WETTREG2010, the statistical model. This model provides ten individual runs per climate scenario analysis which are all plotted in Figure 12 and which show a large variability. Contrary to the other models, WETTREG2010 already overestimated the developments in the past and this overestimation of drought days was more pronounced for the dry sites (Figures 12A–C). In general all models proposed a significant increase in the frequency of the occurrence of drought stress days as compared to simulated mid-last century numbers. The range of this increase was comparable between the dynamic models (REMO, CLM) and all three sites. Irrespective of the type of model used, the increase in the number of days with drought stress was projected to be strongest around the middle of the century and to become less intense at the end of the century. To further understand risks associated with these projections a more in-depth analysis of the year to year variability would be necessary.

## Discussion

The revised and amended model to simulate vineyard water balance is an example for a “sandwich” approach to couple a canopy-based plant water relations model to soil characteristics and climate projections in order to provide a risk assessment for different vineyard sites. The adaptation of the radiation module using a Monte Carlo numerical simulation overcame one of the shortcomings of the original approach of Riou et al. (1989) to treat all horizontal faces of the canopy as opaque which overestimated radiation interception and thus water consumption especially in closely spaced vineyards (Lebon et al., 2003; Figure 4). With this adaptation a new and improved estimation of canopy porosity, *p(x)*, was introduced and experimentally verified which made porosity dependent on the distance a solar beam travels inside of the canopy. This however, will still need adaptation to different canopy forms where leaf area density may be lower than in the VSP systems used in the vineyards of this study, and where thus beam attenuation may follow a different pattern (Poni et al., 1996). Additionally, under prolonged and severe water deficit, leaf drop will increase *p(x)*, and thus reduce transpiration. Nevertheless, sensitivity analyses of previous model versions showed, that a 10% change in *p(x)* only decreased water loss by 1.6% (Trambouze and Voltz, 2001).

At the current state the model does not include stomatal responses to elevated CO_2_ concentrations, which would be important for a more precise impact estimation of future climates on vineyard water relations (Yin, 2013). A general survey of the response of stomatal aperture to an increase to 560 μmol mol^−1^ in CO_2_-concentration (from 380 μmol mol^−1^, Ainsworth and Rogers, 2007) across a variety of plant species showed an approximate reduction of about 20%. Experiments on grapevines have confirmed this value (Schultz and Stoll, 2010) but a reduction in stomatal conductance and possibly the threshold value of *FTSW* to water deficit do not consider possible changes in *VPD* due to climate change. Recent results from models including the physiological impact of CO_2_ on plants (i.e., reduced stomatal conductance) suggest that rising CO_2_ will increase the temperature driven water evaporation from oceans resulting in increased absolute water vapor content of the air. However, the decrease in evapotranspiration over land (because stomatal conductance is decreasing) would still lead to an overall decrease in relative humidity and an increased evaporative demand (Boucher et al., 2009).

Since many vineyard areas in Europe are on slopes with shallow soils more prone to water deficit, the model was adapted to account for the changed radiation budget of sloped vineyards with its consequences on vineyard water relations. A recent study on possible effects of climate change on regional vineyard water budgets did not include inclined surfaces and assumed bare soils (Pieri and Lebon, 2014) which is sufficient for a rough estimate but does not account for large intra-regional variations.

One of the further goals is a scale-up approach to estimate the water budget of entire wine regions based on existing maps of soil water content (i.e., Löhnertz et al., 2004). So far mostly meteorological approaches have been used in studies of climate change effects on Viticulture, where changes in regional water budgets have been either predicted based on extremely rough soil water data with very low spatial resolution (i.e., Malheiro et al., 2010 based on Tonietto and Carbonneau, 2004), used fixed SWC values (Pieri, 2012; Pieri and Lebon, 2014) for all regions, or based their estimations on sub models creating water stress indices which have never been proved to be applicable to vineyard situations (Hannah et al., 2013 based on Alcamo et al., 2003 and Pfister et al., 2009).

We tried to validate the calculated water fluxes through vine canopies by direct measurements of sap flow. However, the model only correlated with sap flow data when a grapevine transpiration coefficient *k*_*c*,*v*_ of 0.56 was introduced. Only then was the seasonal dynamic of *FTSW* accurately simulated. In all previous cases where the original (Lebon et al., 2003) and adapted versions of the model (i.e., Pellegrino et al., 2005; Celette et al., 2010) were compared to measured soil water content (not sap flow), the correlations were excellent without a transpiration coefficient, whether the soil was bare (Lebon et al., 2003; Pellegrino et al., 2005) or had different degrees and/or different types of cover crops (Celette et al., 2010). Explanations for these differences might be that Lebon et al. (2003) used a very low value for the *REW* in their bare soil sub model, possibly indicating an underestimation of bare soil evaporation compensated by an overestimation of grapevine transpiration and Pellegrino et al. (2005) as well as Celette et al. (2010) did their field trials in vineyards with wide row spacings (>2.30 m), where the overall fraction of grapevine transpiration on total evapotranspiration is comparably small (Figure 6). Additionally, as compared to Mediterranean type climates (Pellegrino et al., 2005; Celette et al., 2010) vineyard cover crops in temperate, summer rainfall areas have a larger contribution to whole vineyard evapotranspiration due to less frequent water deficits (Uliarte et al., 2013).

Partitioning of water fluxes between soil, cover crop and grapevines showed that different components dominated during different parts of the season and that soil management had a large impact on flux partitioning. Simulated evaporation levels from soil and transpiration from cover crops were in agreement with direct measurements conducted in the same area (Uliarte et al., 2013) and agreed with those by Celette et al. (2010) for a vineyard with a permanent intercrop and the same row distance when water was not limiting. The rapid decline of bare soil evaporation within a few days after precipitation events simulated by the model was also observed by Uliarte et al. (2013) under similar weather conditions.

The low values in sap flow were surprising but are roughly in line with previous measurements on the same variety (Schmid, 1997). The technique used to measure sap flow was first adopted for grapevines by Braun and Schmid (1999b) and was validated by independent methods at the time (i.e., weighing of large pots). One important restriction of that method, as with any sap flow estimation, is that severe pruning wounds at the trunk can cause large inhomogeneities of the water flux density over the cross sectional area of the trunk (Braun and Schmid, 1999a). This could lead to an overestimation of sap flow if the heating probe is within areas of high flux densities and to an underestimation if the heating probe is located near or in necrotic areas (Schmid, 1997). Since the likelihood of uneven flux density increases with vine age, this may have been part of the reason for the larger confidence intervals of sap flow data for BU and WI, the two older vineyards.

One of the advantages of the formulation of potential grapevine transpiration *T*_0,*v*_ in form of Equation (1) is, that different radiation distributions caused by differences in vineyard geometries and vine training systems are considered and, therefore, ratios for *T*_*a*,*v*_/*T*_0,*v*_ can be directly compared to values from the literature where sap flow has also been estimated. Riou et al. (1994) and Trambouze and Voltz (2001) found ratios which would have been equivalent to *k*_*c*,*v*_ values of 1.25 and 1.12 for a typical vineyard in Bordeaux and 18-year old Shiraz vines in Southern France, respectively. In both cases, the stem heat balance method was used to measure *T*_*a*,*v*_ as described by Valancogne and Nasr (1993). However, Braun and Schmid (1999a) reported that the heat balance system might overestimate actual sap flow by 50–100% at high flow rates in older grapevines. Using heat pulse sensors, Yunusa et al. (2004) found a ratio of *T*_*a*_/*ET*_0_ of 0.17 for non-stressed drip irrigated Sultana vines in Australia. Considering the fractions of shortwave radiation intercepted by the vine canopy for two periods during the growing season in their study resulted in *k*_*c*,*v*_ values of 0.38 and 0.46 which are in the range of our findings. Nevertheless, with the same technique Intrigliolo et al. (2009) found a ratio of *T*_*a*_/*ET*_0_ of 0.49 and a *k*_*c*,*v*_ of 1.6 for 2-year old Riesling vines when sap flow readings were recalibrated with canopy gas exchange measurements (which roughly doubled the calculated transpiration rates). Since sap flow values were similar for all experimental sites under conditions without water deficit in our study and clearly responded to deficit situations, they reflected actual vine responses despite uncertainties with respect to their absolute quantitative accuracy.

One additional aspect of these discrepancies is the large spectrum of stomatal sensitivity to alterations of environmental variables between cultivars and cultivar/rootstock combinations for grapevines (Schultz, 2003; Soar et al., 2006; Williams and Baeza, 2007; Poni et al., 2009; Collins et al., 2010). It is therefore unlikely that a universally valid *k*_*c*,*v*_ exists. Even though *k*_*c*,*v*_ values were similar for the three sites in this study, there were notable differences in the response of *T*_*a*,*v*_/*T*_0,*v*_ to increasing evaporative demand (Figure 8). Although only periods were considered where *a priori* soil water was not limiting (*FTSW* > 0.4), the reduction in *T*_*a*,*v*_/*T*_0,*v*_ with increasing *ET*_0_ for the two drier vineyard sites might have been a response to vapor pressure deficit, *VPD*. High *VPD* in the atmosphere can cause a decline in stomatal conductance in grapevines to control water loss (Soar et al., 2006; Poni et al., 2009; Rogiers et al., 2011) and soil water deficit can exacerbate this response (Soar et al., 2006; Pou et al., 2008; Rogiers et al., 2011; Zhang et al., 2012). Whether this reaction is driven by some factors residing close to the stomatal pores (Peak and Mott, 2011) or depends on hormonal (Soar et al., 2006; Rogiers et al., 2011) or hydraulic long distance signaling (Christmann et al., 2013) inducing stomatal closure is unknown. However, since the driest sites experienced the strongest reduction in the transpiration to evapotranspiration ratio with increasing evaporative demand, it is likely that some form of root-to-shoot signaling was involved. This may have been related to parts of the grapevine root system being located in dry soil, due to inhomogeneous distribution of soil water which has been shown to induce stomatal closure and modulate the response to *VPD* (Poni et al., 2009). Both hormonal and hydraulic limitations have been incorporated into a conceptual water consumption model responsive to *VPD* (Tardieu and Simonneau, 1998) but it seems difficult to fit this into the current framework of the grapevine model although approaches relating the *VPD* response to soil water content parameters similar to the *FTSW* concept may make this possible (Oren et al., 1999; Rogiers et al., 2011). Since *ET*_0_ increases substantially with the degree of slope (Table 5), it is necessary to incorporate these aspects into a more widely applicable model in the future in order to evaluate the propensity of drought risk on a regional scale.

Lebon et al. (2003) also discussed the roles of interception water and surface run-off as possible sources for errors. Run-off is usually negligible for soils with cover crops and small individual precipitation rates, which are dominant in the experimental area (Emde, 1992). To account for the direct interception of water the approach to introduce a skin layer in the bare soil model of Allen (2011) from which water evaporates after precipitation events was applied but that generated only small reductions in soil water content, could not be resolved by the accuracy of the soil water data and was limited to situations where rain fell on dry soils.

As a further adaptation to our climatic conditions, growing stages were introduced to describe the development of the cover crops during the year (Allen et al., 1998). The approach of Celette et al. (2010) to model cover crop development by changes in LAI, was not suitable for our conditions, because the model approach they used (Cros et al., 2003; Duru et al., 2009) did not take into account the destroying impact of frost in cold winters. Calculations assuming that the cover crops are active throughout the year, led to substantial overestimations of vineyard transpiration rates in spring (data not shown).

Additional errors might be introduced by subsurface lateral water flows, because the model does consider vertical flows only. By the occurrence of relief precipitation the variability of rainfall distribution is generally high in regions with slopes. For instance, only a few kilometers north of the Rheingau grape growing region toward the Taunus mountain range the mean annual rainfall is about 250 mm higher. Some but not all soil water access tubes showed an increase of soil water content at certain times in particular layers, which might have been the result of water moving laterally downslope. However, this only occurred during the replenishment stage in winter or spring but not during summer and it cannot be distinguished between vertical or lateral water movements. Also, the increase was restricted to distinct layers and after saturation of the layer the lateral water flow is likely to be through flow. Therefore, the overall error is assumed to be small, but might be an explanation of the underestimation of soil water content by the model for WI in spring.

*FTSW* is strongly correlated with ψ_pd_ (Lebon et al., 2003; Pellegrino et al., 2004; Schultz and Lebon, 2005) which could also be confirmed in the present study (data not shown). This correlation allows the calculation of a water deficit indicator under any environmental situation for scenarios of future climate projections. The approach is appealing since it can serve in several ways to use the model as a tool in climate change research. First, ψ_pd_ can not only be related to physiological processes such as photosynthesis and stomatal conductance but also to the synthesis of grape compositional factors such as anthocyanins and tannins (i.e., Ojeda et al., 2001, 2002). Second, with databases of soil properties, water storage capacities, and rooting depths, available for certain wine regions (Löhnertz et al., 2004), it would be possible to estimate vineyard soil water balance on a regional scale for the next decades. Third, such a model could then be used to identify adaptation possibilities, such as changes in canopy or vineyard characteristics (van Leeuwen et al., 2010), varieties (Schultz and Stoll, 2010) or to recommend/not recommend the installation of irrigation systems (Gaudin and Gary, 2012).

Whereas the dynamic climate models proposed a moderate increase in the number of drought days for all vineyard sites (Figure 12), the statistical model WETTREG2010 projected a much larger effect. This is probably due to the fact, that WETTREG2010 not only projects an increase in temperature and a decrease in precipitation rate, but also a strong increase in global radiation (Kreienkamp, CEC-Potsdam, personal communication) leading to more frequent hot and dry weather conditions during the second half of the century. Nevertheless, the dynamic models CLM and REMO have been shown to be sensitive to the “windward- lee effect,” i.e., an under– or overestimation of precipitation at mountain ranges demonstrated for the South of Germany, thus may have actually underestimated the number of drought days for the Rheingau region (Warrach-Sagi et al., 2013). We have observed such a bias in the precipitation grid data both for runs with the ECHAM5/OM and HadCM3 GCMs.

A risk analysis of probable water shortage in the future can only be as good as the regionalized model predictions of individual meteorological parameters driving the “sandwich” or crop models. Specifically with relation to the future development in summer precipitation and its variability, there is considerable disagreement between individual GCM's (Maraun et al., 2010). Recent analyses of the propensity for drought events in different parts of Europe showed, that the historic patterns observed across Europe were related to shifts in the North Atlantic summer storm tracks which so far are largely unpredictable (Dong et al., 2013).

Despite uncertainties in the projected regionalised precipitation rates, the model will contribute to enlarge the value of more statistical attempts to estimate changes in plant phenology and, thus, the dynamics of grapevine development which is important for water use (Bock et al., 2011; Urhausen et al., 2011).

## Conclusion

We have coupled a soil water balance model with a numerical simulation approach to simulate the distribution of absorbed radiation in vineyards, also accounting for sparse canopies. Sub models, describing the influence of steep slopes, the use of cover crops, and bare soil cultivation on vineyard evapotranspiration were added or replaced to improve the model and simplify its parameterization with the aim to make the model applicable to complete growing regions. The model was validated against soil water and sap flow measurements over two years in three vineyards. Compared to former model approaches, a grapevine transpiration coefficient had to be introduced to accurately simulate measured grapevine transpiration rates. Soil water dynamics in the rooting profile could be adequately described throughout different seasons with different proportions of water loss through bare soil, cover crops or vines. Model runs with data of different RCMs projected an increase of future drought stress occurrence for all sites but varied largely with respect to the absolute number of expected drought days. Similar analyses are needed on a regional scale to develop adaptation scenarios.

### Conflict of interest statement

The authors declare that the research was conducted in the absence of any commercial or financial relationships that could be construed as a potential conflict of interest.

## Appendix A

### Radiation partitioning model

The Monte Carlo simulation serves as a numerical experiment to solve the light partitioning inside the geometry described in Section Radiation partitioning model (Figure 2), where the shortwave radiation is expressed by a random sample of photons (Modest, 2003). The trace of each photon (ray trace) from emission until absorption or reflection back to the sky is followed by calculation of interaction sites, the corresponding probabilities for the possible interactions (transmittance, reflection, or absorption) and random numbers that decide which interactions took place. Therefore, the partitioning of radiation inside the canopy depends on the direction of the incoming radiation beam. This was modelled by a statistically adequate number of equally distributed photons between the rows, emitted between −*D*/2 ≤ *x* ≤ *D*/2, in defined interval steps and with a defined direction at the height *z* = *S* + *H*, where *S* + *H* represents the distance between soil and upper foliage boundary (Figure 2). The interaction sites and possible travel distances inside the foliage were calculated by vector arithmetic in three dimensions. To follow the trace of a photon, it was assumed that absorption and reflection took only place at the bordering faces of the cuboid or the soil surface. Every absorption point of each photon was stored. Random numbers were also used to calculate the directions of diffuse reflections. The surfaces were treated as ideal diffuse reflectors. Literature values were chosen for the shortwave reflectivity (albedo, ρ_*l*_) of leaves (ρ_*l*_ = 0.22) and the soil (ρ_*s*_ = 0.18) (Gates, 1980). No difference was made between green covered or bare soil surfaces at this stage.

To calculate the corresponding probabilities for an interaction of a photon with the grapevine foliage, the transmittance of the foliage was parameterized according to the Beer-Lambert law depending on the porosity (perpendicular to the vertical foliage walls) and the possible travel distance inside the cuboid (Sinoquet and Bonhomme, 1991). If the leaf area distribution is assumed to be homogenous inside the foliage, the porosity *p* can be expressed as:

(A1) $$I(x)/I_{0}=p(x)$$

where *I* is the (non-scattered) radiant flux density, *I*_0_ is the unattenuated flux density and *x* is the distance the radiation travels in the foliage. The equivalent equation based on the Beer-Lambert law is:

(A2) $$I(x)/I_{0}=e^{-kx},$$

where *k* is the extinction coefficient. For the porosity perpendicular to the side walls of the foliage *p*_⊥_ and the corresponding width of the canopy *L*, Equation (A1) becomes:

(A3) $$I(L)/I_{0}=p_{⊥}$$

and the combination of Equations (A1–A3) results in:

(A4) $$\tau (x)=p(x)=\exp(\ln(p_{⊥})\frac{x}{L})=p_{⊥}^{\frac{x}{L}},$$

where τ is the transmittance of the foliage which equals the porosity if the transmittance of single leaves is neglected. For single photons the porosity represents the probability for no interception inside the foliage, i.e., for transmittance (Sinoquet and Bonhomme, 1991). Since the sum of all probabilities for transmittance, reflection, and absorption is unity, it can be written as:

(A5) $$\tau +\rho_{1}(1-\tau )+\alpha_{\nu }=1,$$

where the transmittance τ is determined by Equation (A4), ρ_1_(1 − τ) is the probability that reflection occurs and α_*v*_ is the resulting probability for absorption at the grapevine canopy. In case of interaction at the soil surface, the transmittance is zero. The equation for the probabilities for interactions at the soil surface is:

(A6) $$\rho_{s}+\alpha_{s}=1,$$

where α_*s*_ is the absorptance of the soil surface.

For the purpose of this work only the partitioning between the foliage of the grapevines and the soil was of interest. If the incoming radiation direction is expressed in spherical coordinates (polar angle θ, azimuthal angle φ), the sum *N*(θ, φ) of all emitted photons from the direction θ, φ can be partitioned into photons absorbed by the grapevines (*N*_*v*_), the soil (*N*_*s*_), or reflected back to the sky (*N*_*vy*_). Hence, the interception fractions for direct radiation for grapevines *a*_*v*_, the soil *a*_*s*_ and the albedo ρ_*vy*_ of the vineyard are represented as:

(A7) $$a_{\nu }=\frac{N_{\nu }}{N\left(\theta ,\;\varphi \right)},\;a_{s}=\frac{N_{s}}{N\left(\theta ,\;\varphi \right)},\;\rho_{vy}=\frac{N_{vy}}{N\left(\theta ,\;\varphi \right)}$$

To calculate similar fractions for diffuse solar radiation, it has to be considered that the diffuse irradiance *R*_*dif*_ (in Wm^−2^) received by a horizontal surface is the integral of the radiance *L*_*e*_ (in Wm^−2^sr^−1^) from a solid angle element dΩ over the hemisphere:

(A8) $$R_{dif}=Le\int_{2\pi }\cos\;\theta d\Omega ,$$

where cosθ is the projection from the solid angle element *d*Ω into the horizontal and the radiance *L*_*e*_ is independent of the viewing direction, if the diffuse solar radiation is assumed to be isotropic. To calculate the amount of diffuse radiation which is intercepted by the foliage *R*_*dif*,*v*_, the fractions of direct radiation have to be multiplied with the radiance *L*_*e*_ of the diffuse radiation and integrated over the hemisphere. Expressed in spherical coordinates it follows:

(A9) $$R_{dif,\nu }=R_{dif}a_{dif,\nu }=L_{e}\int_{0}^{2\pi }\int_{0}^{\pi /2}a_{\nu }(\theta ,\;\varphi )\cos\theta \sin\theta \text{d}\theta \text{d}\varphi ,$$

where dΩ = sin θdθdφ is the size of the solid angle element in spherical coordinates and *a*_*dif*,*v*_ is the ratio of diffuse solar radiation which is intercepted by the grapevine canopy. To cover all directions of the radiation from the hemisphere, the numerical experiment provides results for a discrete number of *n* equidistant elements for the intervals 0 < θ < π/2 and 0 < φ < 2π with the interval distances Δθ and Δ φ. Therefore the diffuse fraction index for the foliage could be calculated with Equations (A8) and (A9) and the results of the simulation expressed as:

(A10) $$a_{dif,\nu }=\frac{R_{dif,\nu }}{R_{dif}}=\frac{\sum_{i,j=1}^{n}a(\theta_{i},\;\varphi_{j})\cos\theta_{i}\sin\theta_{i}\Delta \theta \Delta \varphi }{n\sum_{i=1}^{n}\cos\theta_{i}\sin\theta_{i}\Delta \theta \Delta \varphi }.$$

The fractions for direct and diffuse solar radiation thus allow calculating the total radiation absorbed by the canopy *R*_*v*_ or the soil *R*_*s*_:

(A11a) $$R_{\nu }=a_{dif,\nu }R_{dif}+a_{dir,\nu }R_{dir}$$

(A11b) $$R_{s}=a_{dif,s}R_{dif}+a_{dir,s}R_{dir}$$

## Appendix B

### Adaptation of the radiation partitioning model to steep slopes

The partitioning of direct radiation inside the canopy depends on the incoming direction relative to the grapevine rows. Therefore, the position of the sun has to be expressed in a coordinate system which is fixed to the vineyard, the slope system K' (body-fixed frame). The orientation of K' with respect to a horizontal system K (space-fixed frame), can be described by Euler angles which also allow transforming a point from K to K'. The following process was adopted for vineyards with downhill row orientation which simplifies this transformation. The Cartesian coordinate axes of the K system (*x*, *y*, *z*) are defined so that the *z* axis corresponds to the vertical, *y* points to south and *x* to west on the northern hemisphere. The axes of the K' system (*x*', *y*', *z*') are set in order that *z*' corresponds to the normal of the slope system, *y*' points in the direction of the rows and *x*' is in the plane of the slope surface perpendicular to *y*'. If the grapevine rows run downhill (the normal case in German steep slope grape growing regions), and thus *y*' also points downhill, the position of the slope system K' could be defined by two rotations of the K system, with the result that the axes of the rotated system match the *x', y', z*' axes. The first rotation is around the *z* axis with the angle γ and corresponds to the aspect (orientation) of the vineyard. The second is around the *x*' axis (resulting from *x*-axis after the first rotation) with the angle β and corresponds to the slope of the vineyard. Positive rotation directions need to be respected.

The angles β and γ correspond to Euler angles and allow the calculation of the direction cosines, representing the elements of a rotation matrix (Bronstein et al., 1999) with which a point in *x*, *y*, *z* coordinates can be transformed to *x', y', z*' coordinates by:

(B1) $$\begin{array}{l} x^{\prime }=x\cos y\;-\;y\cos\beta \sin\gamma \;+\;z\sin\beta \sin\gamma ,\\ y^{\prime }=x\sin y\;+\;y\cos\beta \cos\gamma \;-\;z\sin\beta \cos\gamma ,\\ z^{\prime }=y\sin\beta \;+\;z\cos\beta . \end{array}$$

The position of the sun is described by the zenith angle θ_*z*_ (the angle between the vertical and the sun), and the solar azimuth angle γ_*s*_ (the angle between the projection of the line to the sun in the horizontal and south, Duffie and Beckman, 2006). To apply Equation (B1) to calculate the position of the sun in the K' system, θ_*z*_ and γ_*s*_ have to be transformed to *x*, *y*, *z* coordinates. The deducted *x', y', z*' coordinates could further be back transformed and expressed as angle of incidence θ (the angle between the sun and the normal of the vineyard) and as vineyard solar azimuth angle γ_*v*_ (the angle between the projection of the line to the sun in the plane of the vineyard surface and the direction of the rows), in analogy to θ_*z*_ and γ_*s*_. Because of the similarity of θ_*z*_ and γ_*s*_ with spherical coordinates this is not outlined here.

## Appendix C

### Estimating direct and diffuse radiation components of solar radiation

We used a correlation between the solar global radiation and a clearness index as described in Duffie and Beckman (2006) to calculate the direct and diffuse radiation components of global radiation. The calculated regression coefficients based on measurements of global and diffuse radiation of the Geisenheim weather station from 2007 to 2012. The diffuse radiation component was measured with a pyranometer with a shading ring. The readings were corrected for the influence of the shading ring by applying correction factors as described by the manufacturer (Kipp and Zonen). For hourly data the correlation was:

(C1) $$\frac{R_{dif}}{R_{glob}}=\{\begin{array}{ll} 1.0-0.065k_{t} & \left(k_{t}\leq 0.22\right)\\ \begin{array}{l} 1.2103-1.9287k_{t}+7.30k_{t}^{2}\\ -16.1542k_{t}^{3}+10.0073k_{t}^{4} \end{array} & \left(0.22<k_{t}\leq 0.8\right)\\ 0.1675 & \left(k_{t}>0.8\right) \end{array}$$

and for daily data:

(C2) $$\frac{R_{dif}}{R_{glob}}=\{\begin{array}{ll} 1.0-0.039k_{t} & \left(k_{t}\leq 0.22\right)\\ \begin{array}{l} 0.8501+2.4950k_{t}-12.2301k_{t}^{2}\\ +16.8298k_{t}^{3}-8.7824k_{t}^{4} \end{array} & \left(0.22<k_{t}\leq 0.8\right)\\ 0.1727 & \left(k_{t}>0.8\right) \end{array}$$

where *k*_*t*_ = *R*_*glob*_/*R*_0_ is the clearness index, defined by the ratio of global radiation, *R*_*glob*_, to extraterrestrial radiation, *R*_0_, and *R*_*dif*_ is the diffuse radiation component.
